# Oral White Lesions: An Updated Clinical Diagnostic Decision Tree

**DOI:** 10.3390/dj7010015

**Published:** 2019-02-07

**Authors:** Hamed Mortazavi, Yaser Safi, Maryam Baharvand, Soudeh Jafari, Fahimeh Anbari, Somayeh Rahmani

**Affiliations:** 1 Oral Medicine Department, School of Dentistry, Shahid Beheshti University of Medical Sciences, Tehran 1983969411, Iran; mortazavi.h@sbmu.ac.ir (H.M.); drjafari@sbmu.ac.ir (S.J.); fahimeh.anbari@sbmu.ac.ir (F.A.); s_rahmani@sbmu.ac.ir (S.R.); 2 Oral and Maxillofacial Radiology Department, School of Dentistry, Shahid Beheshti University of Medical Sciences, Tehran 1983969411, Iran; Safiy2018@sbmu.ac.ir

**Keywords:** mouth disease, oral keratosis, oral leukokeratosis, oral leukoplakia

## Abstract

Diagnosis of oral white lesions might be quite challenging. This review article aimed to introduce a decision tree for oral white lesions according to their clinical features. General search engines and specialized databases including PubMed, PubMed Central, EBSCO, Science Direct, Scopus, Embase, and authenticated textbooks were used to find relevant topics by means of MeSH keywords such as “mouth disease”, “oral keratosis”, “oral leukokeratosis”, and “oral leukoplakia”. Related English-language articles published since 2000 to 2017, including reviews, meta-analyses, and original papers (randomized or nonrandomized clinical trials; prospective or retrospective cohort studies), case reports, and case series about oral diseases were appraised. Upon compilation of data, oral white lesions were categorized into two major groups according to their nature of development: Congenital or acquired lesions and four subgroups: Lesions which can be scraped off or not and lesions with the special pattern or not. In total, more than 20 entities were organized in the form of a decision tree in order to help clinicians establish a logical diagnosis by a stepwise progression method.

## 1. Introduction

Diagnosis of oral white lesions might be quite challenging. These lesions represent a wide spectrum of lesions with different etiology and various prognoses. The diagnosis of white lesions varies from benign reactive lesions to more serious dysplastic and carcinomatous lesions. While there are some classic features that help distinguish these lesions, similar features may give rise to some complications in diagnosis. Efforts should be made to establish a definite diagnosis to prevent time elapse in treatment of patients with more serious lesions. A decision tree is a flowchart that organizes features of lesions in order to help clinicians to reach a logical conclusion. To use the decision tree, one should begin from the left side of the tree, makes the first decision, and proceeds to the far right of the tree where the definite diagnoses are listed [1].

Oral lesions can be classified into four groups comprising of ulcerations, pigmentations, exophytic lesions, and red-white lesions [2]. Although white lesions constitute only 5% of oral pathoses, some of these lesions such as leukoplakia, lichen planus, and proliferative verrucous leukoplakia have malignant potential as high as 0.5–100% [3]. Therefore, white lesions mandate an appropriate clinical diagnostic approach to exclude the possibility of malignancy.

The onset of oral white lesions can be acquired or congenital, with a history of long-lasting existence in the latter form. Oral white lesions can be caused by a thickened keratotic layer or an accumulation of non-keratotic material. Accordingly, when a clinician confronts a white area on the oral mucosa, the first issue to be elucidated is whether it can be scraped off by means of a piece of gauze or not. If so, a superficial non-keratotic layer such as pseudomembranes, most commonly caused by fungal infections or caustic chemicals, should be suspected. Otherwise, white lesions can be attributed to increased thickness of keratin layer, which might have been induced by local frictional irritation, immunologic reactions, or more crucial processes such as premalignant or malignant transformation [4,5].

In the next step, any specific clinical pattern of white lesions such as papular, annular, reticular or erosive-ulcerative patterns, or a combination of them (characteristic for lichenoid lesions) should be inspected in order to differentiate white patterned lesions from non-patterned ones.

Therefore, this narrative review paper focuses on three clinical steps to approach oral white lesions: The first step is to determine whether the lesion is congenital or acquired; the second and third steps are to inspect if it can be wiped off or not and if it has a special pattern or not. This diagnostic process is presented as an updated clinical decision tree. A decision tree is a flowchart used for organizing features of lesions or diseases that help clinicians make a constellation of rational decisions rather than haphazard ones to reach a conclusive diagnosis [1].

## 2. Search Strategy

**Figure 1:**
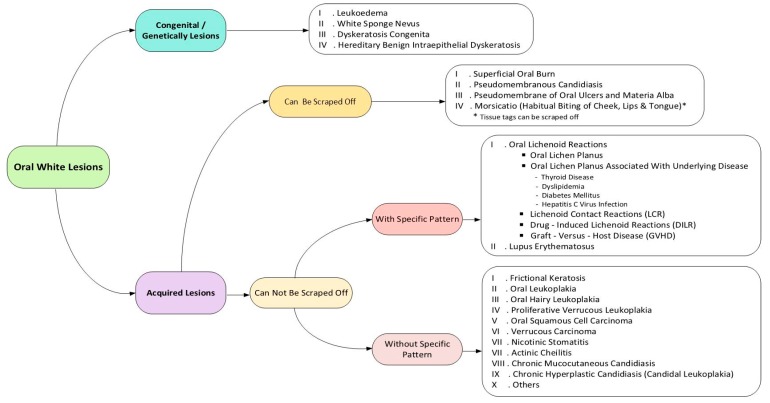
Decision tree of oral white lesions.

General search engines and specialized databases including PubMed, PubMed Central, EBSCO, Science Direct, Scopus, Embase, and authenticated textbooks were used to find relevant topics by means of MeSH keywords such as “mouth disease,” “oral keratosis,” “oral leukokeratosis,” and “oral leukoplakia”. Related English-language articles published from 2000 to 2017 including reviews, meta-analyses, original papers (randomized or non-randomized clinical trials; prospective or retrospective cohort studies), case reports, and case series were reviewed. Out of more than 140 relevant articles and textbooks, five textbooks, and 45 papers were selected including reviews, case reports or case series, and original articles. We described 20 entities, with focus on their clinical aspects. Finally, oral white lesions were categorized into two major groups of congenital and acquired origin and four subgroups: those that can be scrapped off or not, and patterned or non-pattern lesions (Figure 1).

Pictures used in this review article were collected from the archive of Oral Medicine Department of Shahid Beheshti Dental School under permission of the patients by signing the special consent form.

## 3. Congenital/Genetically Lesions

### 3.1. Leukoedema

**Figure 2:**
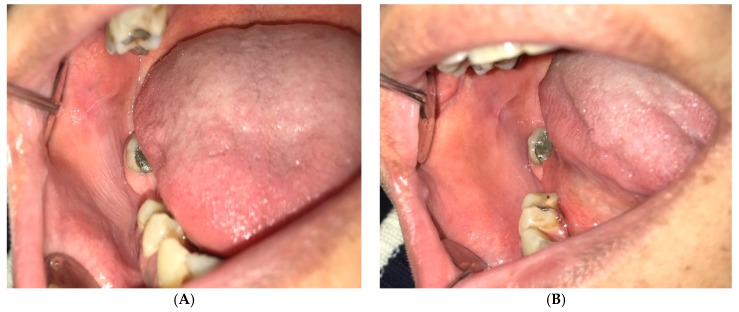
Leukoedema: (**A**) White appearance of buccal mucosa due to Leukoedema. (**B**) By stretching the mucosa, the white wrinkled area disappeared.

Leukoedema is a common normal variation of the oral mucosa. The prevalence has been reported up to 90% among blacks and between 10–50% in whites, with no sex predilection [4,5,6]. Higher prevalence among blacks is possibly due to more mucosal pigmentation making this condition more apparent [5]. It is more distinct in smokers; however, after smoking cessation, it becomes less obvious. It seems to be a developmental variation with an unknown etiology [4,5]. Clinically it presents as a diffuse, gray to white, non-scrapable and veil-like condition, which can be described as a milky and opalescent transformation of the oral mucosa. In more prominent cases, leukoedema is characterized by mucosal folds along with wrinkles or whitish streaks. This condition usually disappears temporarily after gentle stretching of the mucosa, which reappears after quitting the manipulation (Figure 2).

Leukoedema often involves the buccal mucosa and sometimes the lateral borders of the tongue bilaterally. It may spread to the labial mucosa and rarely affects the floor of the mouth, palatopharyngeal and laryngeal tissues [4,5,6,7]. Some extra oral mucosa such as vagina might also be affected [6]. Leukoedema is asymptomatic with no potential for malignant transformation. No treatment is required for this condition [4,5,7] (Table 1).

### 3.2. White Sponge Nevus

White sponge nevus (WSN), also called Cannon disease or familial white folded dysplasia, is an inherited autosomal dominant disorder that is defined as dyskeratotic hyperplasia of mucous membranes [2]. WSN is a rare condition with no sex predilection [8]. A prevalence of below one in 200,000 population has been reported [4]. The lesions generally present at birth or early in childhood, but sometimes the condition appears during adolescence [5,7]. Mutations in keratin genes are responsible for coding of epithelial keratin types K4 and K13 results in lack of normal keratinization [4,5]. Both intraoral and extra oral mucosal sites might be involved. Intraoral lesions are symmetrical, thickened, white, corrugated or velvety, diffuse, spongy plaques of variable sizes with an elevated, irregular, and fissural surface. Buccal mucosa is affected bilaterally in most patients [4,5,7,8]. Other areas of the oral cavity such as the ventral surface of the tongue, labial mucosa, soft palate, alveolar mucosa, and floor of the mouth can also be affected, but the amount of involvement might vary from patient to patient. Extraoral sites include nasal, esophageal, laryngeal, and anogenital mucosa; however, their involvement is relatively unusual in the absence of oral manifestations. White sponge nevus can cause dysphagia when the esophagus is involved; otherwise, the lesions are asymptomatic [4,5]. Due to the benign nature of the lesion, good prognosis, and infrequent recurrence rate no treatment is suggested for WSN [3,4,7] (Table 1).

### 3.3. Dyskeratosis Congenita

Dyskeratosis congenita (DC), also called Coleengman syndrome or Zinsser-Colleengman syndrome, is a bone marrow failure (BMF) syndrome inherited as an X-linked recessive trait with a marked male predilection and women showing less serious clinical manifestations. Infrequent cases of autosomal dominant and autosomal recessive forms have been reported as well [5,9]. Defects in telomere preservation, which protects chromosomal ends against deterioration and inappropriate recombination lead to dyskeratosis congenita [10,11]. This is a rare condition with the annual incidence rate of one per one million population It usually emerges between the ages of 5 to 12 years. Clinical manifestations of DC might be quite various such as abnormal skin pigmentation, nail dystrophy (approximately 90%), oral premalignant leukoplakia (approximately 80%), BMF, cancer susceptibility with elevated risk for squamous cell carcinoma, and hematolymphoid neoplasms [4,9]. Other reported manifestations are intrauterine growth retardation, developmental delay, microcephaly, eyes and hair abnormalities, like premature graying, extreme sweating, short stature, hypogonadism, enteropathy, liver disease, esophageal and urethral stenosis, osteoporosis, and avascular necrosis of the hips and shoulders [9]. The most important oral manifestations are bullae formation followed by erosions, which ultimately progress to leukoplakic lesions in the buccal mucosa, tongue, and oropharynx as well as rapidly progressive periodontal disease, gingival inflammation and bleeding, gingival recession, bone loss, decreased root/crown ratio, and mild taurodontism. The treatment is usually directed to the alleviation of symptoms. Malignant transformation is reported in approximately 30% of the leukoplakic lesions with a progression to Oral Squamous Cell Carcinoma (OSCC) within 10–30 years. Therefore, the physician should schedule frequent monitoring and biopsy taking of suspicious lesions for early diagnosis of potential malignant transformations [4,9,10]. In other words, dyskeratosis congenita is a multi-organ-system disorder that needs regular follow-ups. In severe conditions, patients live approximately 32 years. The patient and the family should receive a genetic consultation. Bone marrow failure is one of the most prevalent and main causes of death, which mandates allogeneic hematopoietic stem cell transplantation as the only main treatment. Less successful treatments such as androgens and oral and topical vitamin E have also been suggested for DC [5,9] (Table 1).

### 3.4. Hereditary Benign Intraepithelial Dyskeratosis

Hereditary benign intraepithelial dyskeratosis (HBID) also called Witkop-Von Sallmann syndrome is a rare autosomal-dominant disorder of the conjunctiva and oral mucosa. This condition primarily reported in descendants of tri-racial isolate (European-American, African-American, and Native American descents) in North Carolina; however, some examples of HBID have been reported sporadically from other areas of the United States, which might be due to migration of affected people. On the other hand, no history of migration to the United States was found in some patients [5,12]. This disorder progresses during childhood with oral manifestations being similar to WSN as thick, corrugated white plaques affecting the buccal and labial mucosa. Milder cases show an opalescent appearance mimicking leukoedema. Other oral mucosal areas such as the floor of the mouth and lateral borders of the tongue might also be involved. Candida species can superimpose on these lesions thereafter. Another clinical manifestation of HBID is ocular lesions presenting as white thick opaque gelatinous plaques on the bulbar conjunctiva adjacent to the cornea with occasional corneal involvement. Eye lesions are found very early in life and increase over time. Patients might complain from tearing, photophobia, and itching of the eyes when the lesions are active. In many cases, the plaques are more obvious in the spring, but they show seasonal regression during summer or autumn. Corneal involvement can result in visual impairment and blindness [5,10,12]. Hereditary benign intraepithelial dyskeratosis is a benign lesion in the oral cavity; hence no treatment modality is needed unless a superimposed infection with candida occurs, which requires antifungal therapy. In case of symptomatic ocular involvement, an ophthalmologist must evaluate the eyes. Generally, eye plaques influencing visual ability should be excised surgically, however these lesions commonly re-appear [5] (Table 1).

## 4. Acquired Lesions That Can Be Scraped Off

### 4.1. Superficial Oral Burn

**Figure 3:**
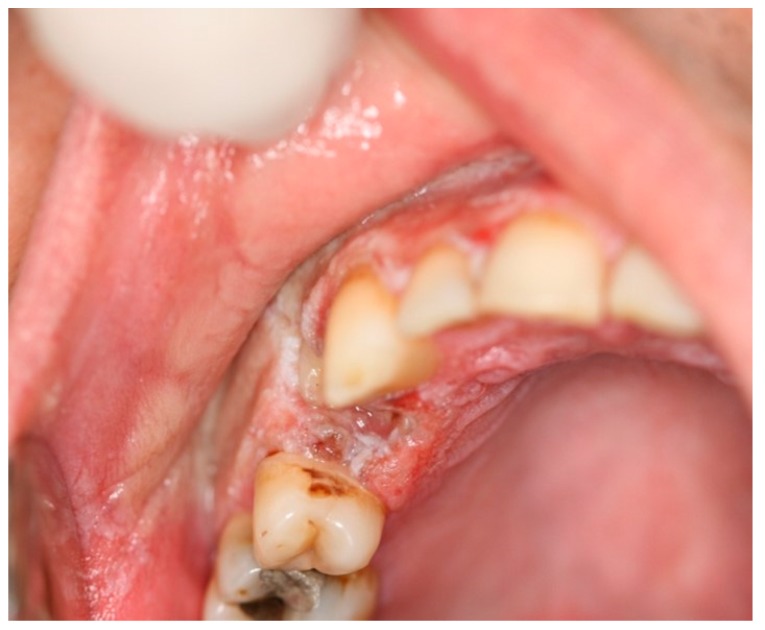
Superficial oral burn due to placement of an over-the-counter anaesthetic gel.

Oral burn includes thermal and chemical burns of the oral cavity. Intraoral thermal burns have been reported frequently, while the chemical injury is not common [13]. Thermal burns of the oral cavity generally result from ingestion of hot foods or beverages like hot pizza or coffee. Extended use of microwave ovens has caused an increased prevalence of thermal burns since they heat up food unevenly in a way that the inner portion of it remains cold while the other part becomes hot [4,5,14]. The most commonly affected areas are palatal mucosa, posterior buccal mucosa, and the anterior part of the tongue. Keratinized mucosa is more resistant to burn than non-keratinized lining mucosa. The extension of injury is related to temperature and duration of contact [5,15]. There is an iatrogenic reason for thermal injury due to accidental contact with hot dental instruments. When the mucosa is anesthetized, the contact with hot instruments might continue longer, which results in more extensive burn injury [4]. On the other hand, chemical burns can result from the use of chemical materials like topical applications of medications to relieve dental pain (Figure 3).

Some of well-documented caustic materials are aspirin, sodium perborate, hydrogen peroxide, gasoline, turpentine, rubbing alcohol, battery acid, isopropyl alcohol, phenol, eugenol, carbamide peroxidase, bisphosphonates, chlorpromazine, and promazine [5,13]. Thermal burn usually is mild and affects just a small area as a sloughy yellow-white necrotic epithelium with areas of erythema and ulceration [5,13,14]. However, chemical burns result in mucosal necrosis with more severe clinical features [13]. In case of short-time exposure to chemicals, the superficial mucosa becomes white and wrinkled, but longer exposures lead to denudation of the epithelial layer and development of a yellowish, fibrinopurulent membrane over the area [5]. Most cases of mucosal burns have little clinical effects and improve without treatment [5,13]. Use of rubber dam is a preventive technique in order to decrease iatrogenic mucosal burns [5]. According to the size and symptoms of the lesions, some recommendations are proposed to manage these conditions such as use of non-steroidal anti-inflammatory drugs (NSAID’s), antibiotics, antiseptic mouthwashes, coverage with a protective emollient paste or a hydroxypropyl cellulose film, topical anesthetics, and surgical debridement [5,15] (Table 2).

### 4.2. Pseudomembranous Candidiasis

**Figure 4:**
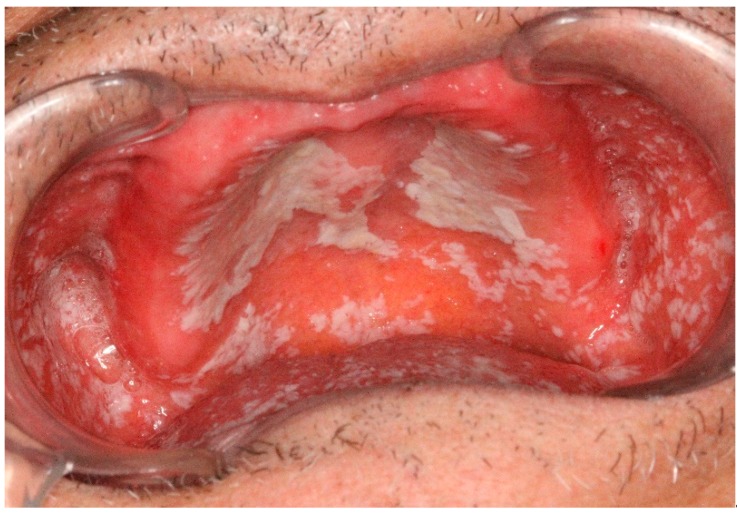
Pseudomembranous candidiasis due to using broad spectrum antibiotic; pseudo membranes can be scraped off by a piece of gauze.

Oral candidiasis is the most common fungal infection of the oral cavity mostly caused by *Candida Albicans* as one of the normal microflora organisms [4,16]. It can be isolated from almost 50% of dentate patients and more than 60% of edentulous individuals with a female predilection [4]. The acute pseudomembranous form of oral candidiasis is usually seen in infants due to their underdeveloped immune system, the elderly because of their compromised immunity and patients on broad-spectrum antibiotics [4,17] (Figure 4).

It presents as creamy white plaques, patches, or papules that can be wiped off with an erythematous and sometimes bleeding area leaving behind [4,5,17]. The classic appearance of pseudomembranous candidiasis is called “curdled milk” [5]. Burning sensation and foul taste might accompany the lesions as well [5]. The chronic pseudomembranous oral candidiasis is not distinguishable from its acute counterpart that emerges in patients with HIV infection and those taking corticosteroid inhalers [4]. The buccal mucosa is frequently affected by pseudomembranous candidiasis followed by the tongue and the palate. Elimination of predisposing factors, if possible, is the cornerstone of treatment along with antifungal regimen [4] (Table 2).

### 4.3. Pseudomembrane of Oral Ulcers and Materia Alba

An epithelial defect in the ulcerative process is usually coated by a pseudomembrane composed of necrotic cells and fibrin. It is usually seen in aphthous ulcers, erythema multiforme, and other ulcerative conditions of the oral cavity. The color of a fibrin clot is white, dirty yellow-white, or grayish-white [4,18].

Materia Alba is defined as accumulated oral debris resulting from poor oral hygiene forming a plaque-like appearance such as a coated tongue. Sometimes this condition is misdiagnosed with other pathologic white lesions. Both pseudomembrane and material alba can be easily wiped off. Under the pseudomembrane, a raw, bleeding, and painful surface appears while wiping materia alba leaves a quite normal mucosa underneath [18,19,20] (Table 2).

### 4.4. Morsicatio

**Figure 5:**
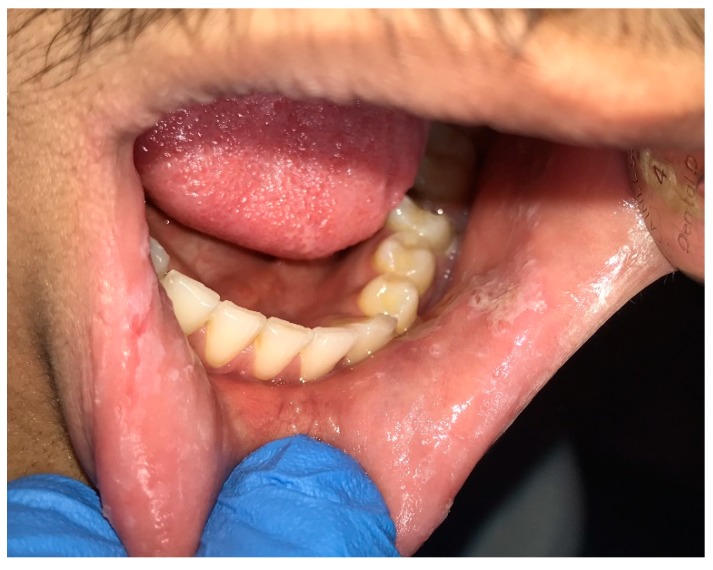
Habitual biting of cheeks and the lower lip.

Morsicatio originates from the Latin word *morsus*, meaning “bite”, which also called morsicatio mucosa oris or chronic mucosal chewing [5]. This lesion is caused by self-induced injury and chronic tissue irritation like habitual chewing of buccal mucosa, chronic nibbling, biting, or sucking (Figure 5).

Most patients deny the self-inflicted injury or do it subconsciously. Glass blowers develop similar changes in their buccal mucosa due to chronic irritation as well. The prevalence of morsicatio has been reported 0.5% to 1.12% among the general population with a male to female ratio of 1/3. This condition is more common in patients older than 35 years, and those having extra stress or mental illness [4,5,21]. The most affected areas are non-keratinized epithelium of the buccal mucosa (morsicatio buccarum), lips (morsicatio labiorum), and the lateral borders of the tongue (morsicatio linguarum), respectively. Morsicatio does not involve areas not achievable to habitual chewing trauma [4,5,14,21]. While the lesions usually present bilaterally on the mid-portion of anterior buccal mucosa along the occlusal line extensive lesions may involve larger areas of buccal mucosa. Sometimes morsicatio might appear as unilateral lip and/or tongue lesions [5]. The clinical features include asymptomatic shaggy and thickened macerated gray-white patches or plaques with keratin shreds, tissue tags, or desquamated areas on the mucosal surface, which gradually merge the adjacent mucosa [4,14,21]. A peeling irregular ragged surface with erythema or erosion—but not ulceration—might accompany white areas, and the patient may report the ability to peel shreds of the white portion from the periphery of the lesions [4,5,21]. If the clinical presentation of morsicatio is typical and history taking reveals patients’ habit of mucosal biting the diagnosis will be established. In case of any doubt, a biopsy seems to be necessary [4,5,21]. This condition has no long-term negative consequences and generally no treatment is recommended. As morsicatio usually occurs subconsciously, the patients should give consultation for their parafunctional behavior to resolve it. Nonetheless in some patients whose chewing habit is difficult to quit use of night guard has been suggested to eliminate the injury of adjacent teeth to oral mucosa [4,5,14] (Table 2).

## 5. Acquired Lesions That Cannot Be Scraped Off (With Specific Pattern)

### 5.1. Lichenoid Reactions

Lichenoid reactions represent a family of lesions with different etiologies, but the same clinical and histologic appearance. Neither clinical nor histopathologic features enable the clinicians to discriminate between different lichenoid reactions [4] (Table 3).

### 5.2. Oral Lichen Planus

**Figure 6:**
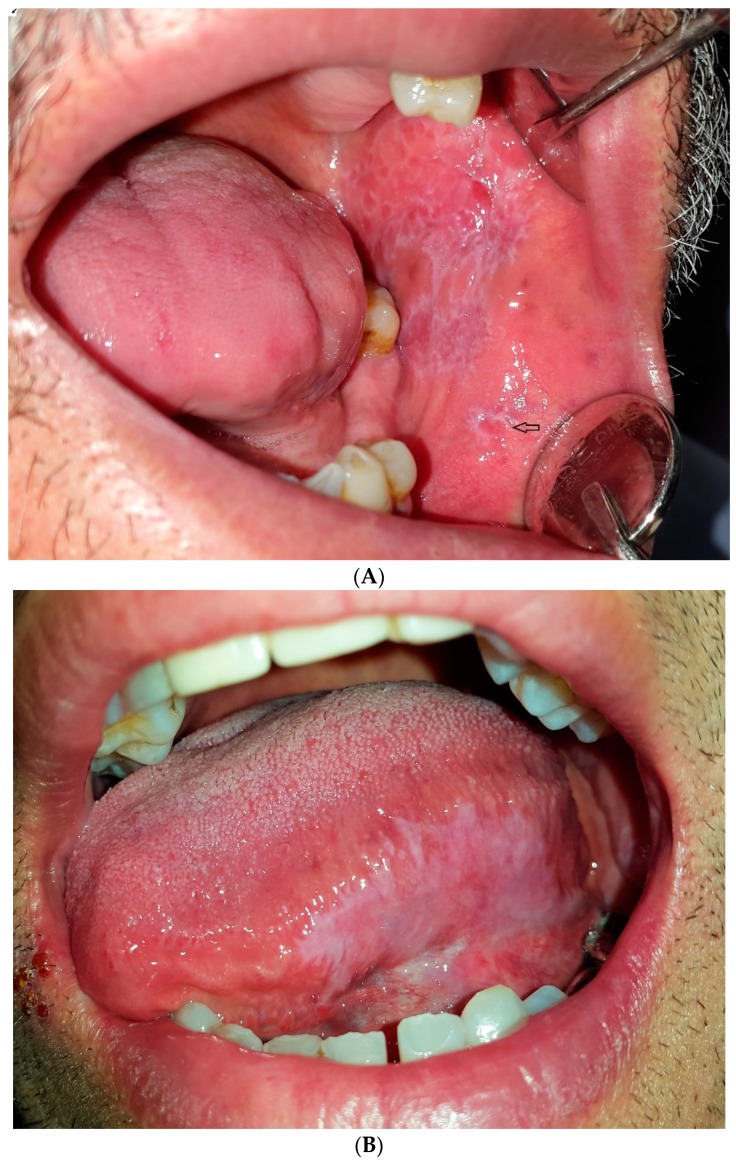
Clinical features of oral lichen planus with specific white pattern, (**A**) reticular OLP; the arrow shows annular form; (**B**) plaque-like OLP.

Lichen planus (LP) is defined as a common chronic mucocutaneous disease with unknown etiology. Oral lichen planus (OLP) involves 0.1–2.2% of general population and mostly arises after middle age with a mean age of 55 years. Women are affected more frequently than men with a female to male ratio of 3:2 [4,5,22]. The mostly affected extra oral mucosal site is the genitalia. Cutaneous lesions can be detected in approximately 15% of patients [4,5,22]. Although the etiology is multifactorial, imbalanced immune system with the presence of autoreactive T lymphocytes plays a principal role in the evolution of this disease [4]. Other factors such as stress have also been noticed in the development of this inflammatory process [4,7]. Oral lichen planus has various clinical manifestations including papular, reticular, plaque-like, bullous, erythematous, and ulcerative features [4,22]. White components might be seen as papules, plaques, and reticular areas [17]. Generally, the papular type of OLP is found in the primary phase of the disease. Then, small white papules are usually combined together to form the reticular pattern. Fine white lines or striae (also mentioned as *Wickham’s striae*) comprise the reticular feature of OLP, which might form a network or annular (circular) form (Figure 6).

Frequently, the striae accompany a surrounding erythematous area. Reticular OLP often affects the posterior buccal mucosa bilaterally, sometimes the lateral and dorsal tongue, the gingiva, the palate, and infrequently the mucosal side and vermilion border of the lips [4,5,7,22]. Plaque-type OLP appears as a homogeneous well-demarcated white plaque with peripheral striae. OLP lesions on the dorsum of the tongue become clear as keratotic white plaques with glossitis without any striae [4,5]. Erythematous (atrophic), bullous, and ulcerative forms of OLP are less common and often surrounded by a white reticular network. Sometimes the erythematous OLP involves attached gingivae without any papules or striae, which is called desquamative gingivitis. The reticular, papular, and plaque-like forms of OLP commonly present without any symptoms, except for a brief roughness. Patients with the erythematous form of OLP feel a burning sensation during eating, however, the most debilitating form of OLP is ulcerative type [4,5,17]. The presence of papules or reticular elements helps clinicians establish an accurate clinical diagnosis. These characteristic components may be obvious in conjunction with plaque-like, erythematous, bullous, or ulcerative lesions. A biopsy is mandatory in gingival erythematous lesions with no obvious striae or papules to achieve a correct diagnosis [4,17]. Reticular symptomless type of OLP requires no treatment. In some patients superimposition of *Candida* species may cause a burning feeling of the oral mucosa, which makes the use of antifungal agents necessary. Erosive lichen planus often has symptoms such as pain and burning. A topical steroid with concurrent use of antifungal drugs is the first line of management. Systemic steroids are advised to reduce symptoms of resistant lesions. Other proposed treatments include calcineurin inhibitors, retinoids, and ultraviolet phototherapy [4,5,7,17,22,23]. OLP is considered as a potentially malignant disorder with a low risk (approximately 0.2% per year) of malignant transformation to OSCC. Therefore, precise annual monitoring of these patients is advocated [4,5,22,24]. It has been reported that plaques, erosive and ulcerative sites, especially on the soft palate, lateral, and ventral surface of the tongue or floor of the mouth show more tendency for malignant transformation, and biopsy should be considered to exclude dysplasia or carcinoma [4,5,17].

### 5.3. Oral Lichen Planus Associated with Underlying Diseases

Some systemic diseases and medical conditions copy the same clinical appearance of OLP. Recent studies have noticed the relationship between OLP and hepatitis C virus in some populations such as Central and Eastern Asia, Middle East, North Africa with high prevalence (over 3.5%), South Asia, sub-Saharan Africa, Central and Latin America, the Caribbean, Oceania, Australia, Central and Eastern Europe, Western Europe with medium prevalence (1.5–3.5%), and North America, Northern Europe with low prevalence (below 1.5%) [11,25]. Genetic divergence has been considered as an explanation for these differences [1,5]. Dyslipidemia is another condition which reported being significantly associated with OLP with a prevalence of 58% in OLP patients. It is shown that chronic inflammation results in disruptions in the lipid metabolism like reducing high-density lipoproteins–cholesterol (HDL-C), increasing very low-density lipoprotein-cholesterol (VLDL-C) and hypertriglyceridemia [26,27]. In addition, a relationship between thyroid disease and OLP especially hypothyroidism has been proposed. Significantly increased amounts of serum anti-thyroglobulin, autoantibodies and anti-thyroid microsomal autoantibodies were found in these patients. The prevalence of thyroid disease in patients with OLP has been reported 2.19% to 6.46% [28]. Moreover, OLP has been found to have a major correlation with diabetes mellitus (DM), which might be due to endocrine dysfunction and immunological defects. A recent meta-analysis study showed the prevalence of OLP was 1.37% in DM patients and 0.75% in control subjects [29].

### 5.4. Lichenoid Contact Reactions

**Figure 7:**
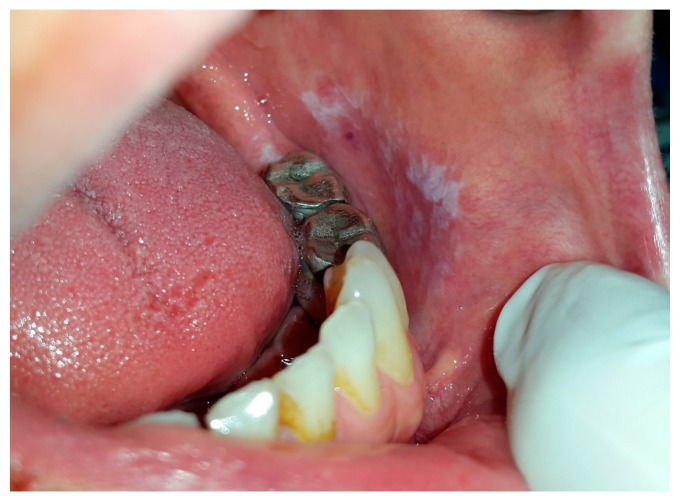
Lichenoid contact reaction in buccal mucosa and alveolar ridge due to amalgam build-up of 1st and 2nd mandibular molar.

Lichenoid Contact Reactions are considered to be a delayed hypersensitivity reaction to constituents derived from dental materials. Since the majority of patients show a positive patch-test to mercury, LCR is considered an allergic reaction. Nearly all dental restorative materials, except for precious metals such as titanium, palladium, and zirconium might give rise to LCRs [4,5,30] (Figure 7).

No prevalence rate has been reported for LCR yet. Women are affected more frequently than men. Clinically, LCRs show the same features as seen in OLP, that is, reticulum, papules, plaques, erythema, and ulcers [4,5]. The most apparent clinical difference between OLP and LCR is the extension of the lesions. The majority of LCRs are confined to sites just in contact with dental materials, such as the buccal mucosa and the lateral borders of the tongue. Lesions are hardly ever observed in sites such as the gingivae, palatal mucosa, floor of the mouth, or dorsum of the tongue. Most LCRs are non-symptomatic, but the patient might experience discomfort from spicy and hot food when the lesion converts to erythematous or ulcerative forms. The duration of contact with dental material has the key role to develop LCRs on the oral mucosa. Lichenoid reactions caused by dental composites have been observed on the mucosal side of both upper and lower lips. Replacement of dental materials in direct contact with LCR will result in cure or considerable improvement in at least 90% of the cases within one to two months [4,5,30]. However, it is not necessary to replace restorative materials that are not in direct contact with LCRs. While a malignant potential for LCRs has been suggested, no prospective studies have been conducted to support this hypothesis [4].

### 5.5. Drug-Induced Lichenoid Reactions

Drug-Induced Lichenoid Reactions (DILRs) are related to a delayed hypersensitivity reaction. It has been suggested that drugs or their metabolites precipitate the lichenoid reaction. DILRs are uncommon and account for a very low percentage of this entity [4]. There are many drugs responsible for the development of these lesions such as non-steroidal anti-inflammatory drugs and angiotensin-converting enzyme inhibitors. Other medications, such as anti-malarial and antihypertensive drugs, diuretics, oral hypoglycemic agents, gold salts, penicillamine, and beta blockers are also related to the evolution of DILRs [30]. DILRs are mostly unilateral with an ulcerative pattern, which might be quite similar to OLP. Usually the lesions evolve several months after the patient starts a new drug. DILRs are not generally severe; however, if a patient has serious symptoms withdrawal of the drug and use of topical steroids are often recommended [4].

### 5.6. Graft-Versus-Host Disease (GVHD)

Graft-versus-host disease (GVHD) is a major complication of allogeneic hematopoietic cell transplantation. Oral GVHD mimics clinical features of OLP, but with a more generalized distribution and concomitant involvement of other organs such as skin and liver. Oral manifestations of GVHD are found in 25–70% of the patients, which might appear as the only clinical feature of GVHD [3]. It is characterized by lichenoid inflammation that can involve all intraoral sites, but particularly affects the tongue, buccal mucosa and lips. Clinical signs ranged from only mild reticulation to more extensive disease with painful ulcerations. The soft palate is infrequently affected and it rarely extends posteriorly to the oropharynx. Treatment of GVHD, especially when there is a multisystem involvement requires systemic corticosteroids and/or other immunomodulatory agents [31].

### 5.7. Lupus Erythematosus

Lupus erythematosus (LE) is an autoimmune disease classified into systemic lupus erythematosus (SLE), chronic cutaneous lupus erythematosus (CCLE), and subacute cutaneous lupus erythematosus (SCLE). SLE is a multisystem disorder with oral involvement, while CCLE involves the skin and oral mucosa. The clinical manifestations of SCLE are intermediate compared to the aforementioned types [5]. The prevalence of LE in the United States is more than 1.5 million patients. SLE affects nearly 1 in every 2000 people with a female to male ratio of 9:1 and the mean age of 31 years at the time of diagnosis. The etiology remains unknown, however increased action of B lymphocytes and autoantibody production with the imbalanced function of T lymphocytes have been identified. Genetic and environmental factors such as infections mostly with EBV and other viruses, contact with pollutants, hormonal factors, ultraviolet light, smoking, diet, and use of some drugs are the predisposing factors for this condition [4,5]. There is an extensive range of clinical symptoms for SLE. Skin lesions (85%) include characteristic butterfly rash (40–50%), alopecia, photosensitivity, Raynaud’s phenomenon, livedo reticularis, urticaria, erythema, telangiectasia’s and, cutaneous vasculitis. Sunlight often aggravates the malar rash [4,5]. Involvement of kidneys (50–60%), musculoskeletal system (95%), central nervous system (CNS) (20%) and cardiovascular system, coagulation disorders, fatigue, depression and fibromyalgia-like symptoms, serositis, gastrointestinal and ophtalmic disorders have also been reported [4]. Oral manifestations (9–45% in SLE, 3–20% in CCLE) include ulcerations, erythematous lesions, hyperkeratosis, honeycomb plaques, and discoid lesions. The lesions generally involve the palate, buccal mucosa, and gingivae. Sometimes, vermilion zone of the lower lip (lupus cheilitis) is also affected [4,5,32]. Ulcers are often aphthous-like with a white to yellow coating and a peripheral red rim especially in the hard palate [32]. A honeycomb plaque is a rare condition, revealed as a chronic, well-defined plaque along with white lacy hyperkeratosis and buccal erythema. The lesions generally affect both lining and masticatory mucosa, however they are less hyperkeratotic on the lining mucosa (e.g., soft palate). Discoid oral lesions appear as whitish steriae generally radiating from the central erythematous area (“brush border” pattern), which makes it difficult to distinguish them from oral candidiasis or OLP if there is no systemic or cutaneous findings [4]. Lupus cheilitis is an inflammatory condition of the lips presenting as a small or diffuse, erythematous and edematous lesion that might develop into crusty painful ulcers. This condition usually affects the vermilion zone of the lower lip [32]. Oral manifestations of CCLE are similar to erosive OLP with an ulcerated or atrophic, erythematous central area and peripheral white, fine, radiating striae. Occasionally the central region shows a fine stippling of white dots along with erythema. However, the oral features are generally accompanied with skin lesions. When ulcerative and atrophic oral lesions come into contact to acidic or salty foods pain might arise similar to erosive OLP. Oral features of SCLE are the same as those of CCLE [4]. Diagnosis of SLE can be quite challenging in primary stages because of its ambiguous clinical course usually with remission phases. American Rheumatism Association has set some clinical and laboratory criteria for the diagnosis of SLE [5]. Occasionally, radiating white striae of oral lesions resemble Wickham’s striae of OLP; Therefore, biopsy is required for definite diagnosis [32]. Mild cases can be successfully managed by means of NSAIDs along with anti-malarial agents. Systemic corticosteroids in combination with other immunosuppressive agents and immunomodulators are frequently used for more severe conditions. Meanwhile, systemic therapy would lead to the amelioration of oral lesions if any [5] (Table 3).

## 6. Acquired Lesions That Cannot Be Scraped Off (without Specific Pattern)

### 6.1. Frictional Keratosis

Frictional (traumatic) keratosis is defined as white plaques with a rough and frayed surface clearly related to an identifiable source of mechanical irritation. These lesions can occasionally mimic dysplastic leukoplakia. The prevalence has been reported as high as 5.5%. This category includes linea alba, and cheek, lip, and tongue chewing. Traumatic keratosis has never been shown to undergo malignant changes. Once the irritant is removed the lesion must resolve within two weeks; otherwise, biopsy is mandatory to rule out a dysplastic lesion [6].

### 6.2. Oral Leukoplakia

**Figure 8:**
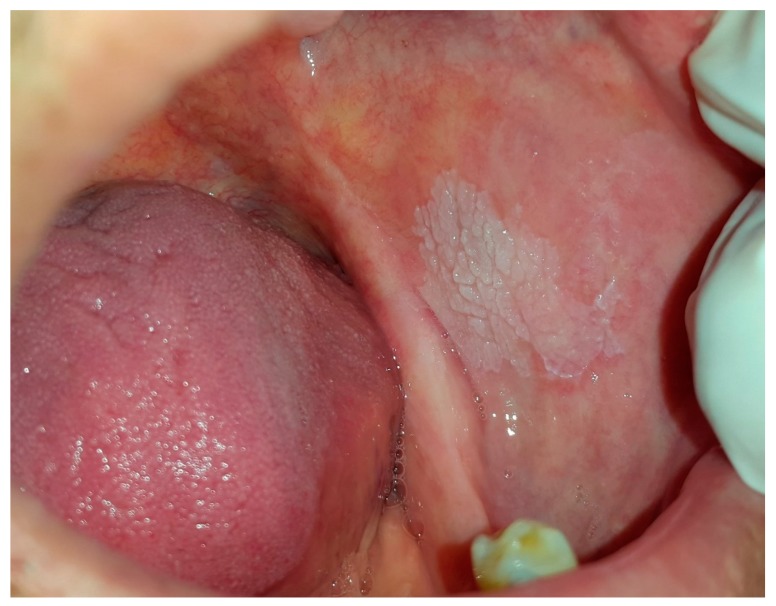
Leukoplakia on the buccal mucosa.

Oral leukoplakia (OL) has been defined as a white patch or plaque that cannot be attributed to any clinically or histologically definite lesion [22,33]. The prevalence of OL is reported 2.6% among general population. Most lesions are seen above the age of 50 with men being more commonly affected; however, a slight predilection for women has been found in some studies [4]. OL is the most frequent potentially malignant lesion of the oral mucosa in a way that 16% to 62% of oral squamous carcinoma are related to a pre-existing leukoplakia. While the etiology of these lesions remained unexplained some authors mentioned the relationship between leukoplakia and tobacco, alcohol, sanguinaria, ultraviolet radiation, trauma, betel quid chewing, genetic factors, and microorganisms [4,5,33,34]. Clinically, OL manifests as an irreversible, non- scrapable and slightly raised white plaque that may have a wrinkled, leathery to “dry or cracked-mud” appearance. These lesions are divided into homogenous or non-homogenous types. The homogenous type has a regular, smooth whitish surface and well-defined margins. The non-homogenous form of leukoplakia consists of an erythematous part (erythroleukoplakia or speckled type) or a nodular, erosive, ulcerated, or verrucous exophytic component. In the speckled type the lesion is predominantly white. The verrucous leukoplakia has an elevated, proliferative, or corrugated surface, and the nodular type develops small polypoid enlargements or rounded mostly white excrescences [7,22,34] (Figure 8).

Oral leukoplakia is generally localized or widespread on the buccal mucosa, lip vermilion, and gingivae. Suggested management includes the elimination of predisposing factors, use of beta-carotene, lycopene, ascorbic acid, α-Tocopherol (Vitamin E), topical and systemic retinoic acid (Vitamin A), topical bleomycin, cold-knife surgical excision, laser surgery along with regular follow up [3,7,17] (Table 4).

### 6.3. Oral Hairy Leukoplakia

**Figure 9:**
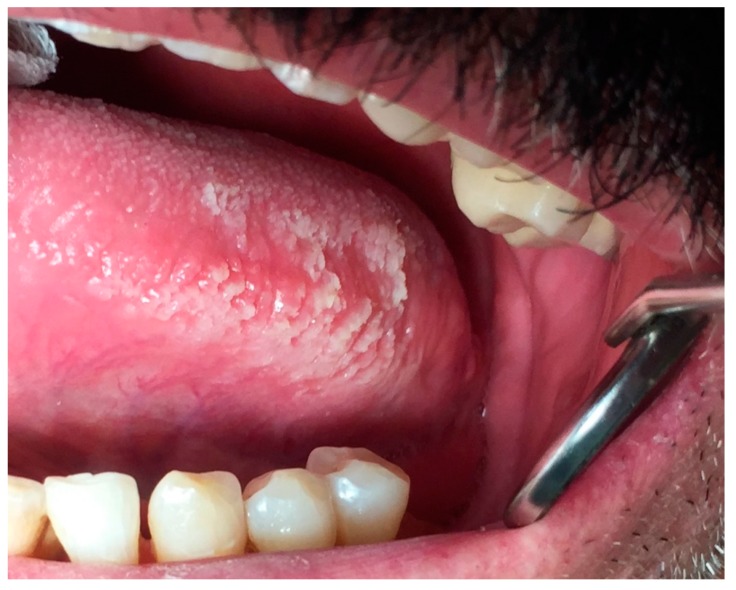
Oral hairy leukoplakia on the lateral border of the tongue with vertical white folds.

Oral hairy leukoplakia (OHL) is a white lesion that develops in immunosuppressed patients infected with Epstein-Barr Virus or having low levels of CD4 + T lymphocytes. OHL is an indicator of progress towards AIDS stage in HIV infection, but it might occur in other states of immune deficiencies and very rarely in immune competent individuals [4,5,17,34]. Highly active antiretroviral therapy has reduced the prevalence of OHL significantly, however, in patients with AIDS the prevalence rises to 80%. OHL is more commonly observed among men with no potential for malignant transformation [4,17]. It is clinically described as whitish nonscrapable velvety plaques that symmetrically involve the borders of the tongue unilaterally or bilaterally. The shape of plaques varies from slight, white vertical bands to thickened, furrowed areas with a shaggy surface [5,34,35] (Figure 9).

OHL infrequently spread to cover the entire dorsal and lateral surfaces of the tongue. Rarely, the buccal mucosa, soft palate, pharynx, or esophagus can be affected [5]. While oral hairy leukoplakia is asymptomatic, superimposed infection with candida create symptoms of mild pain and taste alteration [4,35]. Treatment of OHL generally is not needed, and it has been reported to display spontaneous regression; however minor discomfort or esthetic concerns may require therapy. Therapeutic interventions include systemic anti-herpes virus drugs, topical retinoids or podophyllum resin, combination therapy with acyclovir cream and podophyllum resin, gentian violet, surgical excision, or cryotherapy [5,35] (Table 4).

### 6.4. Proliferative Verrucous Leukoplakia

**Figure 10:**
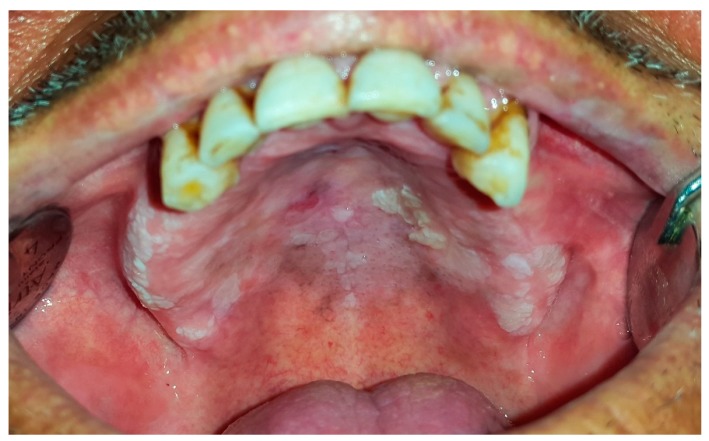
Proliferative verrucous leukoplakia spreading over hard palate and alveolar ridges.

Proliferative verrucous leukoplakia (PVL) is a different and threatening form of OL. The WHO has defined it as ‘‘a rare but distinctive high-risk clinical form of oral precancerous lesions” [22,36]. Proliferative verrucous leukoplakia commonly appears in the elderly women with no racial preference. The mean age of patients with long-lasting lesions of PVL was reported over 60 years [4,5,34,37]. While the etiology of this condition is uncertain genetic factors and viral infections such as *Human Papilloma Virus* especially type 16 and 18 and *Epstein-Barr Virus* have been proposed [36,37]. Clinical appearance in the early stage contains small whitish and well-defined patches or plaques appearing as focal and homogeneous keratotic lesions (Figure 10).

The lesions enlarge slowly and constantly over time involving diffuse surfaces of the mucosa. Meanwhile, non-homogeneous multifocal areas with speckled and rough surface might appear in the form of exophytic, wart-like, verrucous, polypoid projections or erythematous features [5,34,36,37]. PVL usually develops bilaterally, which affects the buccal mucosa, gingivae, and alveolar ridges. The gingivae have been reported as the most affected area; hence a PVL subtype named proliferative verrucous leukoplakia of the gingivae has been proposed [22,36,37]. The rate of malignant transformation for PVL is reported between 63.3% to 100%. It might eventually progress to develop OSCC or verrucous carcinoma [22,36]. Various treatments modalities such as surgery, carbon dioxide laser ablation, topical photodynamic therapy, oral retinoids, topical bleomycin solution, beta-carotene, methisoprinol (a synthetic antiviral agent), radiation, and chemotherapy are suggested. PVL is a refractory condition with a recurrence rate of 85%. None of the treatment methods is effective in quiting relapses and malignant transformation, and therefore a lifelong follow-up is mandatory [36,37].

### 6.5. Oral Squamous Cell Carcinoma

**Figure 11:**
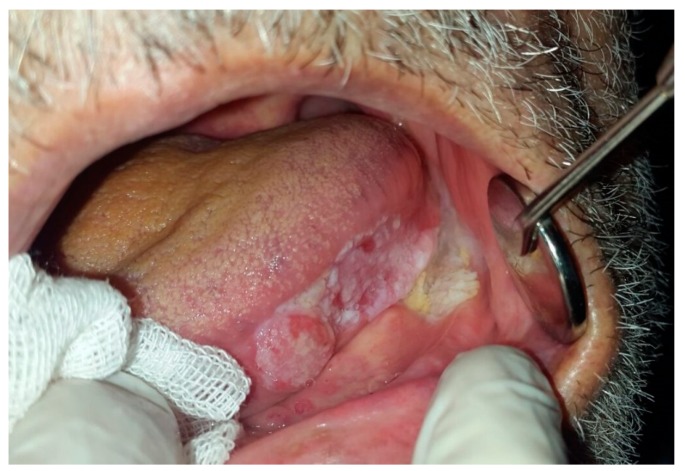
Squamous Cell Carcinoma with verrucous, plaque like and exophytic clinical manifestations at the lateral border of the tongue, extending to the floor of the mouth.

Oral squamous cell carcinoma (OSCC) comprises 92–95% of all oral cancers [22]. The incidence has been reported higher among men and patients older than 65 years. Etiology of OSCC is multifactorial including tobacco smoke, alcohol consumption, betel quid, phenol, viral, bacterial and fungal infections, electro-galvanic reaction, radiation, genetics, immunosuppression, expression of oncogenes, deactivation of tumor suppressor genes, malnutrition, iron-deficiency anemia, and some heritable conditions [4,5,22]. Oral lesions may present as red, white, or combined red-and-white lesions; alteration of the surface texture into granular, rough, fungating, papillary, and verruciform or crusted lesion may be seen; a mass or irregular ulceration with rolled border and induration on palpation may also exist (Figure 11).

The lesion can be flat or elevated with some palpability or induration. The Floor of the mouth, posterior lateral borders and ventral surface of the tongue are considered high-risk areas for OSCC. Involvement of submandibular and digastric lymph nodes due to cancer causes lymphadenopathy with firm to hard consistency, which converts to fixed nodes in later stages [4,5]. Patients are most often diagnosed in advanced phases after progression of symptoms related to disease. Five-year survival rate of OSCC is about 53–56% [22]. Treatment planning depends on clinical staging; therefore, primary stages are treated by surgical process and advanced cases may be managed by radiation therapy or combined chemoradiation therapy with or without surgery [5] (Table 4).

### 6.6. Verrucous Carcinoma

Verrucous carcinoma (Ackerman’s tumor, snuff dipper’s cancer, Buschke-Loewenstein tumor, florid oral papillomatosis, epithelium acuniculatum, carcinoma cuniculatum) is a rare subtype of oral squamous cell carcinoma with distinct clinical and histopathological features [38,39] comprising 2%–9% of all oral carcinomas [40]. It is found mostly in the elderly men over 55 [5]. Oral verrucous carcinoma usually shows slow growth rate, local invasion, and a low tendency to metastasize. However, these characteristics depend highly on the size of the tumor and the time of diagnosis [38,39]. Some precursor lesions such as leukoplakia, erythroplakia, and proliferative verrucous leukoplakia can evolve to verrucous carcinoma over time [38]. Its etiology is not well understood, but some causative habits like smoking, alcohol consumption, betel nut chewing, and smokeless tobacco have been postulated [5,38]. The role of *Human Papilloma Virus* in the oncogenesis of verrucous carcinoma has yet to be elucidated [38]. Verrucous carcinoma manifests as an asymptomatic, diffuse, well-demarcated, thick white plaque with papillary or verruciform projections on the surface [5,38]. Sometimes the lesion tends to be pink in coloration due to an inflammatory reaction to the tumor. All parts of oral mucosa can be affected, however mandibular vestibule, buccal mucosa, gingivae, tongue, and hard palate are the most involved sites. In case of tobacco dipping in the maxillary vestibule or floor of the mouth, these locations are affected more frequently [5].

A malignant transformation has been related to oral verrucous carcinoma. It comprises less than 1% to 16% of OSCC with an annual incidence rate of one–three cases per million [1]. It is reported that the rate of malignant transformation in gingival lesions is about 21 times higher than tongue lesions [38]. Surgery is considered the treatment of choice in most cases; however, combination therapy with surgery and radiotherapy is preferred in extensive lesions. The risk of recurrence rises when surgery or radiotherapy is implemented alone [5,38,39] (Table 4).

### 6.7. Nicotinic Stomatitis

**Figure 12:**
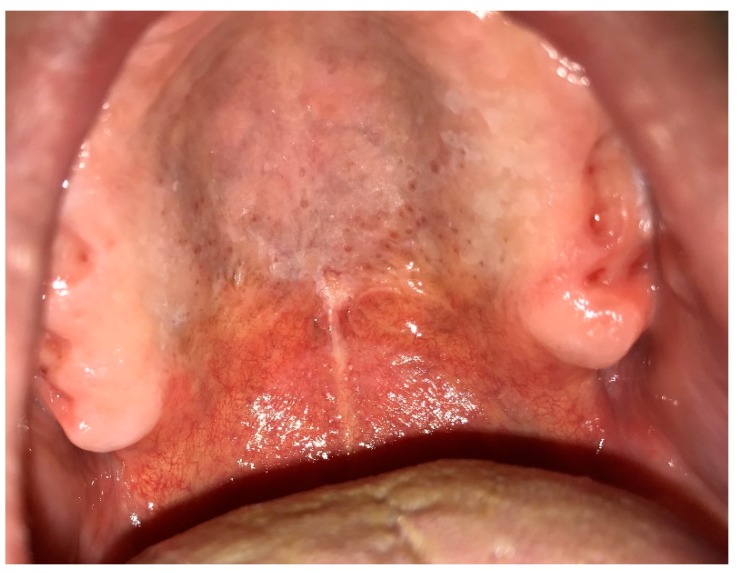
Nicotinic stomatitis on the hard palate.

Nicotine stomatitis is a usual white lesion due to smoking, which is also called Nicotine Palatines or smoker’s palate [4,5]. This condition is commonly seen among heavy smokers of pipe, tobacco, cigarettes, and reverse smokers as well as patients who drink extremely hot beverages habitually. A frequency of 0.1% to 2.5% has been reported with a predilection for men older than 45 years [4,5,7]. Albeit both high temperature and chemical ingredients of smoke have synergistic effects on developing nicotine stomatitis, the impact of high temperature is much higher than that of chemicals. Nicotine stomatitis primarily presents as an erythematous area on the posterior rugae; then the lesion converts to diffuse leathery grayish-white palatal plaque. Red points can also be seen on the white mucosa that are actually widened and swollen orifices of accessory salivary glands with periductal nodular keratinization (Figure 12).

In addition, thickened palatal mucosa intermingled with fissures produce a “dried mud” appearance. White plaques may affect marginal gingivae and interdental papillae as well [4,5,7,41]. Nicotine stomatitis can entirely regress after cessation of smoking and be replaced with normal mucosa. This is not a premalignant condition; however, reverse smoker’s palate has a considerable potential for malignant transformation. Therefore, any white lesion of the palatal mucosa lasting longer than one month after habit cessation should be carefully monitored to rule out malignancy [5,7] (Table 4).

### 6.8. Actinic Cheilitis

Actinic Cheilitis (AC) is a chronic inflammatory condition also called actinic cheilosis or solar cheilosis [5,42]. The basis of etiopathogenesis is UV light exposure; however other risk factors such as old age, fair complexion, immunosuppression, arsenic exposure, and genetic abnormalities are also implicated. A significant male predilection with a male to female ratio of 10:1 might be attributed to more outdoor occupational activities among men. Therefore, sometimes the terms like “farmers’ lip” and “lip of sailors” are used. Primary clinical features are dryness, swelling, and cracks with smooth, blotchy, pale atrophic regions on the lower lip vermilion (95%) [5,22,42]. Moreover, the border between vermilion and the adjacent skin cannot be easily distinguished. The lesions progress to rough, scaly, crusting areas and keratotic plaques on drier segments of the vermilion. Finally, chronic ulceration appears, and persists for months and progresses to malignant lesions [5,42]. Some predisposing factors such as immunosuppression and tobacco smoking can convert AC to a malignant condition (SCC) in 6% to 10% of patients. The warning signs of bleeding, induration, recurrence and sustained pain are suggestive of transforming AC to SCC. Various treatments such as surgery, cryotherapy, electrosurgery, topical retinoids, 5-fluorouracil cream, photodynamic therapy, CO_2_ laser ablation, and vermilionectomy have been suggested for AC [5,22,42] (Table 4).

### 6.9. Chronic Mucocutaneous Candidiasis

Chronic mucocutaneous candidiasis (CMC) is described as a recurrent or consistent disease involving the nails, skin, oral, and genital mucosa initiated by *Candida spp*., most frequently *C. Albicans* [43]. CMC is a rare clinical condition that begins during infancy in 60%–80% of patients, therefore the late onset is uncommon [44]. The etiology is associated with hereditary or acquired T-cell deficits and alteration in the gene responsible for producing cytokine IL-17 that is related to mucosal immunity against *Candida Albicans* [5,43]. Chronic mucocutaneous candidiasis appears as whitish plaques, along with crusts and ulcers frequently found in the oral and pharyngeal mucosa as well as gastrointestinal and vaginal mucosa [44]. These thick, white plaques of oral lesions generally cannot be scraped off similar to chronic hyperplastic candidiasis, however other clinical forms of candidiasis may appear as well [4]. Various antifungal medications have been suggested for the treatment of oral lesions with some degrees of resistance to antifungal therapy. Imidazole derivatives such as ketoconazole, fluconazole, and itraconazole can be used to manage CMC [5,44].

### 6.10. Chronic Hyperplastic Candidiasis (Candidal Leukoplakia)

Chronic hyperplastic candidiasis (CHC) also called as candidal leukoplakia, chronic plaque-type and nodular candidiasis is the least common form of oral candidiasis presenting as white patches or plaques which cannot be detached upon scraping and cannot be attributed to any other lesions [4,5,22,45]. Chronic hyperplastic candidiasis constitutes almost 7%–50% of all oral leukoplakias [46]. It is mainly a disease of adulthood with the age range between 31–81 years, and the majority of patients are over 50 [46]. Chronic hyperplastic candidiasis is frequently presented as well-demarcated, palpable, raised lesions from small translucent whitish areas to large opaque plaques. If the plaque has a smooth, homogenous white surface, it is called a homogenous leukoplakia [46]. However, the surface often has a fine intermixture of red and white areas resembling a speckled leukoplakia usually possessing a nodular component [5,46]. Chronic hyperplastic candidiasis is typically located on the retro commissure areas bilaterally [4,46]. The tongue, palate, and lips can also be involved [4,5,22,45]. Whether it is simply a candida infection superimposed on a preexisting oral leukoplakia or a newly formed leukoplakia induced by candida species is a matter of debate [5]. An increased risk of malignancy related to CHC has been found as compared to normal mucosa [46,47]. Moreover, a four–five times risk of malignancy has been found in comparison to leukoplakia not associated with candidal infection [22]. Several local or systemic predisposing factors have been identified for CHC such as lack of mucosal integrity, wearing dentures, hyposalivation, acidic and high-glucose-content saliva, tobacco smoking or chewing, diabetes mellitus, immunodeficiency, deficiency of iron and folic acid, high-carbohydrate regimen, and non-secretor status of blood group antigen [46]. Different modalities have been proposed to treat CHC with various success rates such as antifungal therapy, topical application of retinoids, betacarotene, bleomycin, several surgical techniques like cold-knife surgery, laser therapy, and cryosurgery. Many clinicians prefer to treat the lesions with antifungals prior to surgical methods [46,48].

## 7. Others

Whiteness or pallor of oral mucosa can be seen in some uncommon conditions such as submucous fibrosis, some granulomatous diseases, skin grafts, scar, and uremic stomititis [4,6,19,49,50].

## 8. Discussions

White lesions of the oral cavity constitute a wide variety of entities with different pathogenesis and clinical features. We proposed a decision tree to classify such lesions according to their clinical manifestations. This helps clinicians to make a more accurate differential diagnoses list.

The first major group, congenital non-scrapable white lesions of the oral cavity (Table 1), most commonly appear early in the life with a history of familial involvement [10,11,12]. When the white plaque fades with stretching leukoedema should be suspected, especially in a smoker patient with the involvement of buccal mucosa [4,11]. On the other hand, diffuse white plaques in the oral cavity along with extraoral mucosal lesions are in accordance with white sponge nevus [8]. Oral white plaques accompanied by conjunctival plaques and eye lesions are usually seen in patients with hereditary benign intraepithelial dyskeratosis [13]. Similarly, dyskeratosis congenita appears as oral white lesions concomitant with nail dystrophy [9,11].

The second major group of oral white lesions is acquired lesions, which can be scraped off (Table 2). Some of the lesions in this category such as mucosal burns, morsicatio, and pseudomembranes of ulcers are due to trauma and can be easily diagnosed by detection of the insulting factor on history taking and clinical examination [4,5,13,14,15,21]. While pseudomembranous candidiasis in adults and the elderly suggests a systemic or local predisposing factor like debilitating disease or oral microflora imbalance it is quite common in infants and considered somehow normal [4,16]. Noteworthy, scraping a pseudomembrane covering an oral ulcerative lesion will result in a bleeding surface, but in case of candidiasis punctuated bleeding will appear. However, mucosa under derbies has normal appearance [4].

Oral aquired white lesions, which are keratotic and cannot be scraped off are further divided into lesions with specific clinical pattern and those without any specific feature. Because of the profound similarity of keratotic lesions with no clinical pattern, it is mandatory to consider some minor characteristics to differentiate them.

The third major group of oral white lesions is white keratotic lesions with a specific pattern (Table 3), which can be differentiated from other entities by their special clinical features like discrete papules, annular or reticular forms; however, they are not distinguishable from each other nor clinically neither microscopically. This group comprises of lichenoid reactions (oral lichen planus, lichenoid contact reaction, drug-induced lichenoid reaction, graft versus host disease) [27,28,29,30]. Presence of papules or reticular elements with symmetrical and generalized distribution along with skin or extraoral mucosal (genitalia) involvement would be in favor of lichen planus [4,30]. On the other hand, LCRs usually develop on the mucosa adjacent to a dental restoration or appliance [5,30]. Drug-induced lichenoid reactions (DILRs) are mostly unilateral with a positive history of taking medications capable of inducing such lesions. In addition, resolving of the lesions upon withdrawal of the drug and reappearance or exacerbation by re-challenging confirms the diagnosis of lichenoid drug reaction [30]. Oral GVHD lesions are more widespread than OLP among patients with a past medical history of hematopoietic stem cell transplantation and sometimes with concomitant involvement of other organs such as skin and liver [31]. Another lesion with a clinical specific pattern similar to lichenoid lesions is chronic cutaneous lupus erythematosus (CCLE) with a discoid-patterned lesions comprising of a central ulcerated, atrophic, or erythematous area with white, fine, radiating steriae at the periphery, which is more prominent than OLP and may abruptly terminate with a sharp demarcation [32].

The fourth subgroup of oral white lesions is keratotic lesions that cannot be scraped off without a specific pattern (Table 4). The clinical key for diagnosis of frictional keratosis is accordance of the suspected lesion to the site of chronic mechanical trauma. The traumatic factor can be detected either clinically (e.g., fractured tooth, ill-fit denture, etc.) or through history taking (e.g., self-inflicted trauma) [14,20]. Presence of a keratotic plaque with punctuated interspersed red dots on the palate with a history of smoking or drinking hot beverages are diagnostic for nicotinic stomatitis [4,5,41]. Actinic cheilitis is suspected when white lesions are seen on the lips among patients with a prolonged history of sun exposure like farmers, fishermen, and other out-doors workers [42]. Chronic hyperplastic candidiasis is considered in patients with underlying disease and simultaneous cutaneous and mucosal candida lesions. A symmetrical white plaque with pigmentation usually with a cracked-mud appearance in the retro commisural area in a smoker patient is suggestive of chronic hyperplastic candidiasis [46,47,48]. Rapid growth in combination with various clinical features such as ulceration, tissue necrosis, and surface non-homogeneity would prompt the clinician to consider SCC in the upper rankings of differential diagnosis [5,22,40]. Despite SCC, verrucous carcinoma cannot metastatize. It is associated with smokeless tobacco and often exhibits a rough surface [38,39,40]. Oral leukoplakia is diagnosed when other keratotic white lesions are excluded clinically and histopathologically [33,34]. Proliferative verrucous leukoplakia is suspected when a slowly progressive multifocal leukoplakia with an uneven surface is encountered in a female patient who is not a smoker [36,37]. White vertical bands with a shaggy surface involving borders of the tongue unilaterally or bilaterally in an immunosuppressed patient are characteristic of oral hairy leukoplakia [35].

## 9. Conclusions

White lesions of the oral cavity include some critical entities that need an organized approach for making the correct diagnosis. In this article, an updated decision tree was proposed in order to help clinicians to make timely differential diagnoses through a stepwise progression method.

## Figures and Tables

**Table 1:** Characteristics of congenital/genetically white lesions.

Entity	Age	Gender	Common Location	Clinical Features	Treatment	Premalignant
Leukoedema	Not Assigned (NA)	M = F	buccal	mucosal folds, wrinkled white strerias	NA	NA
White sponge nevus	presents at birth	NA	buccal, ventral surface of the tongue, labial mucosa, soft palate, alveolar mucosa, floor of the mouth	symmetrical, thickened, white, corrugated or velvety, diffuse spongy plaques with an elevated, irregular and fissural surface	NA	NA
Dyskeratosis congenita	5–12 years	NA	buccal, tongue, oropharynx	bullae formation, erosions, leukoplakic lesions, rapidly progressive periodontal disease, gingival inflammation and bleeding, gingival recession, bone loss, decreased root/crown ratio, mild taurodontism	bone marrow transplantation (BMT), androgens, oral and topical vitamin E	30% malignant transformation in leukoplakia
Hereditary benign intra epithelial dyskeratosis	childhood	NA	buccal, labial	opalescent appearance mimicking leukoedema/thick, corrugated white plaques	NA	NA

**Table 2:** Characteristics of acquired white lesions that can be scraped off.

Entity	Age	Gender	Common Location	Clinical Features	Treatment	Premalignant
Superficial burn	NA	NA	palatal, posterior buccal, anterior tongue	sloughy yellow-white necrotic epithelium with the areas of erythema and ulceration	NSAIDs, antiseptics, antibiotics, analgesics	NA
psuedomembranous candidiasis	infants/elderly	F > M	buccal, tongue, palate	creamy white plaques, patches, or papules	antifungals	NA
Pseudomembrane of oral ulcers and materia alba	NA	NA	NA	a white, dirty yellow-white or grayish-white color	removal of the debris, oral rinses	NA
Morsicatio	>35 years	F > M	buccal, lips, lateral border of the tongue	shaggy and thickened macerated gray-white patches or plaques with keratin shreds, tissue tags or desquamated areas	cessation the habitual chewing	NA

**Table 3:** Characteristics of acquired white lesions that cannot be scraped off, with specific pattern.

Entity	Age	Gender	Common Location	Clinical Features	Treatment	Premalignant
Lichen planus	middle age, mean age of 55	F > M	posterior buccal mucosa bilaterally	papular, reticular, plaque-like, bullous, erythematous and ulcerative features; white components might be seen as papules, plaques, and reticular areas	topical steroid, concurrent use of antifungal drugs	potentially malignant disorder
LCR	NA	F	restricted to sites that are regularly in contact with dental materials such as buccal mucosa and lateral borders of the tongue	same reaction patterns as seen in OLP, that is, reticulum, papules, plaque, erythema, and ulcers	replacement of dental materials	NA
DILR	NA	NA	NA	unilateral with an ulcerative reaction pattern	withdrawal of the drug and use of topical steroids	NA
GVHD	NA	NA	tongue and buccal mucosa	hyperkeratotic reticulations and plaques, erythematous changes, and ulcerations	systemic corticosteroids and/or other immunomodulatory agents	NA
SLE	mean age: 31	F	palate, buccal mucosa, and gingivae	ulcerations, erythematous lesions, hyperkeratosis, honeycomb plaque and discoid lesions as whitish steriae generally radiating from the central erythematous area (brush border)	NSAIDs along with antimalarial agents, systemic corticosteroids in combination with other immunosuppressives and immune modulating agents	NA

**Table 4:** Characteristics of acquired white lesions that cannot be scraped off, without specific pattern.

Entity	Age	Gender	Common Location(s)	Clinical Features	Treatment	Premalignant
Frictional keratosis	NA	NA	NA	white plaque with rough and frayed surface	removal of irritants	NA
Oral leukoplakia	>50 years	M	buccal mucosa, lip vermilion and gingivae	white patch or plaque	NA	potentially malignant lesion
OHL	NA	M	borders of the tongue unilaterally or bilaterally	from slight, white vertical bands to thickened, furrowed areas with a shaggy surface	systemic anti-herpes virus drugs, topical retinoids or podophyllum resin, combination therapy with acyclovir cream and podophyllumresin, gentian violet, surgical excision or cryotherapy	no potential for malignant transformation
PVL	mean age: 60 years	F	gingivae	non-homogeneous multifocal areas with speckled and rough surface in the form of exophytic, wart-like, verrucous, polypoid projections or erythematous components	surgery, carbon dioxide laser ablation, topical photodynamic therapy, oral retinoids, topical bleomycin solution, beta-carotene, methisoprinol (a synthetic antiviral agent), radiation, chemotherapy	malignant transformation
OSCC	>65 years	M	floor of the mouth, posterior lateral borders and ventral surface of the tongue	red, white, or combined red-and-white lesion; alteration of surface texture into granular, rough, fungating, papillary, and verruciform or crusted lesion; or existence of a mass or irregular ulceration with rolled border and induration on palpation.	radiation therapy or combined chemo radiation therapy with or without surgery	NA
Verrucous carcinoma	elderly	M	mandibular vestibule, buccal mucosa, gingivae, tongue, and hard palate	asymptomatic, diffuse, well demarcated, thick white plaque with papillary or verruciform projections	NA	NA
Nicotinic stomatitis	>45	M	palate	diffuse leathery grayish-white palatal plaque with red points, “dried mud” appearance	regression after cessation of smoking	not a premalignant condition
Actinic cheilitis	old age	M	lower lip vermilion	dryness, swelling, cracks, atrophic regions, crusting regions, keratotic plaques, chronic ulceration	Surgery, cryotherapy, electrosurgery, topical retinoids, 5-flurouracil cream, photodynamic therapy, CO_2_ laser ablation and vermilionectomy	Premalignant condition
Chronic mucocutaneous candidiasis	begins during infancy	NA	nails, skin, oral and genital mucosae	chronic whitish plaques, along with crusts and ulcers	antifungal therapy	NA
chronic hyperplastic candidiasis (Candidal leukoplakia)	over 50	NA	retro commisures bilaterally, tongue, palate and lips	white patches or plaques	antifungal therapy, topical retinoids, betacarotene, bleomyin, several surgical techniques	NA
